# Hidden Webs and Small Bowel Obstruction: Non-steroidal Anti-inflammatory Drug (NSAID)-Induced Diaphragmatic Strictures

**DOI:** 10.7759/cureus.101692

**Published:** 2026-01-16

**Authors:** Rhett L Harmon, Nicholas Cheung, Robin Dietz Jr., Shaun Chandna, Payman Fathizadeh

**Affiliations:** 1 Internal Medicine, Olive View-UCLA (University of California Los Angeles) Medical Center, Sylmar, USA; 2 Pathology, Olive View-UCLA (University of California Los Angeles) Medical Center, Sylmar, USA; 3 Gastroenterology, Olive View-UCLA (University of California Los Angeles) Medical Center, Sylmar, USA

**Keywords:** explorative laparotomy, non-steroidal anti-inflammatory drug (nsaid), non-steroidal anti-inflammatory medicines (nsaids), small bowel diaphragm disease, small bowel enteropathy, small bowel resection

## Abstract

Diaphragm disease is an uncommon manifestation of non-steroidal anti-inflammatory drug (NSAID)-associated enteropathy, in which thin, concentric fibrotic septa constrict the small bowel lumen, most often in the distal ileum. Because the septa are typically <2 mm thick and radiolucent, patients are frequently misdiagnosed with adhesive small bowel obstruction (SBO) and may undergo unnecessary laparotomy. We present a case of an 89-year-old man with prolonged, high-dose ibuprofen use for degenerative knee pain who developed complete SBO. CT enterography showed a distal-ileal transition point, and exploratory laparotomy revealed three circumferential diaphragms 30 cm proximal to the ileocecal valve. Microscopic examination of multiple luminal narrowings revealed radially arranged submucosal fibrovascular proliferation lined by mucosa with scattered acute injury and repair changes, characteristic of NSAID-related diaphragm disease. Segmental resection relieved the obstruction, and the patient recovered uneventfully following permanent cessation of NSAIDs.

## Introduction

Small bowel obstruction (SBO) accounts for approximately 15% of emergency surgical admissions, with adhesions responsible for two-thirds of presentations [1]. When obstruction develops in patients with no history of abdominal surgery, a broader differential, including tumors, hernia, Crohn’s disease, gallstone ileus, bezoar, and medication-related strictures, should be considered [2].

Among the world’s most widely consumed drugs are non-steroidal anti-inflammatory drugs (NSAIDs). Injury to the upper gastrointestinal mucosa is commonly attributed to the chronic use of NSAIDs; much less commonly described are instances of lower gastrointestinal tract disease, which often escapes detection until complications arise [3]. Besides the classic finding of NSAID-induced erosions and ulcers, enteropathy may also manifest as diaphragm disease, a pattern of short, circumferential webs resembling a thin ring that commonly arises in the ileum and less often in the jejunum [4]. Incidence of diaphragm disease in long-term NSAID users is approximately 2%, with presenting symptoms of gastrointestinal obstruction such as abdominal pain, nausea and vomiting (72.9%), followed by gastrointestinal bleeding (65.8%), and less common symptoms of weight loss (18.1%) and diarrhea (14.2%); very rarely do patients present with acute perforation (2.6%) [5]. Pathogenesis is multifactorial: topical mucosal injury from enterohepatic bile-NSAID conjugates, mitochondrial oxidative stress, inhibition of prostaglandin-mediated perfusion, and cyclical ulceration and healing with chronic, concentric collagen deposition have all been proposed as contributing factors [6,7]. The average duration of NSAID-associated diaphragm disease has been reported as seven years [8]. Recognition has improved with capsule and balloon-assisted enteroscopy, yet the diagnosis is often missed on routine computed tomography due to limitations in visualization.

## Case presentation

An 89-year-old man with hypertension and hypothyroidism presented with three days of colicky abdominal pain, nausea without vomiting, and complete cessation of flatus. He had been self-medicating with ibuprofen 800 mg three times daily for 14 months for degenerative knee pain. He denied previous abdominal surgery, recent unintentional weight loss, inflammatory bowel disease, or gastrointestinal bleeding. On arrival, he was afebrile with blood pressure at 154/70 mmHg, heart rate at 65 beats per minute, and oxygen saturation at 98% on room air. Abdominal exam revealed tympany and mild epigastric and right lower quadrant tenderness without guarding. Laboratory studies revealed WBC 9.3 cells/µL (reference range: 4.5-10.0 cells/µL), hemoglobin 12.6 g/dL (13.5-16.5 g/dL), ferritin 18 ng/mL (22-275 ng/mL), transferrin saturation 9% (20%-50%), and lactate level 1.1 mmol/L (0.5-2.2 mmol/L).

Imaging

**Figure 1:**
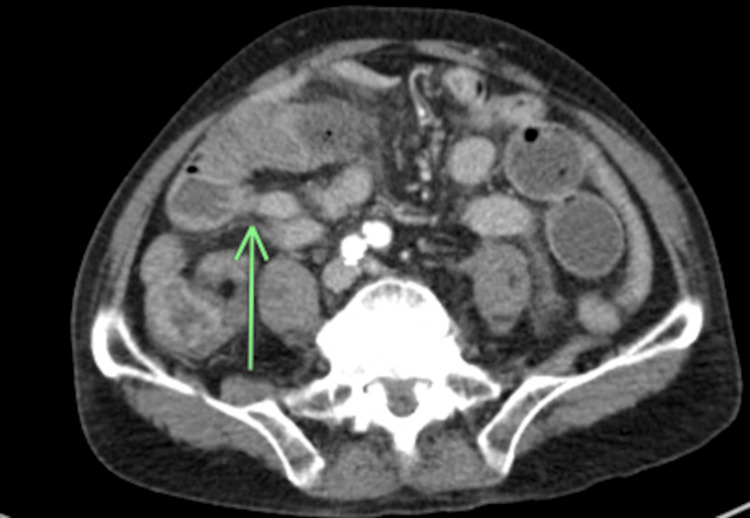
Axial CT of the abdomen and pelvis demonstrating moderate- to high-grade small bowel obstruction There is fecalization to the level of a transition point (green arrow) in the distal small bowel in the right lower quadrant. There is diffuse distension with dilated segments of the small bowel. No pneumatosis or free air was seen.

**Figure 2:**
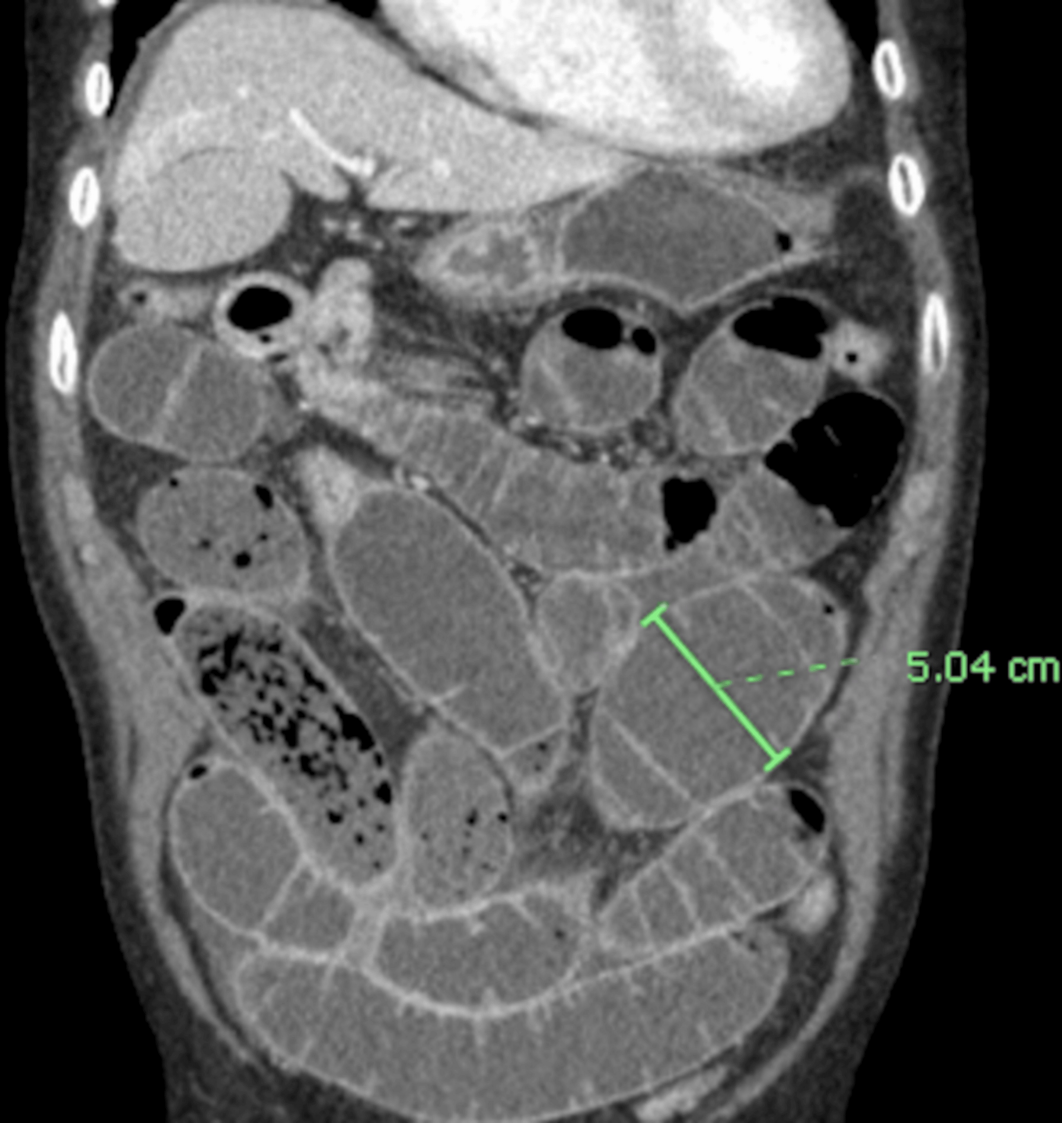
Coronal CT enterography imaging showing diffuse small bowel dilatation The small bowel demonstrates increased distension throughout, with a dilated segment measuring over 5 cm. No pneumatosis or free air is seen.

Abdomen/pelvis CT with contrast was performed, which demonstrated mid-small bowel dilation to 3.3 cm with a single transition point in the right lower quadrant, no free air, and no mesenteric stranding (Figure 1). CT enterography demonstrated comparatively worsened small bowel dilatation over 5 cm, likely secondary to contrast ingestion; no mural thickening, hyperenhancement, mesenteric fat stranding, or additional strictures were identified (Figure 2).

Operative management

After 48 hours of nasogastric decompression and intravenous fluids, the obstruction persisted, prompting exploratory laparotomy on hospital day 3. Intraoperative findings were notable for SBO secondary to a distal ileal stricture and phytobezoar approximately 30 cm proximal to the ileocecal valve. An enterotomy was performed, resulting in the removal of the phytobezoar and evacuation of 2 L of intestinal contents. A few additional sites of mild structuring proximal to the primary site of obstruction were also found. The remainder of the bowel was normal. A 40 cm segmental ileal resection with primary anastomosis was performed.

Pathology

**Figure 3:**
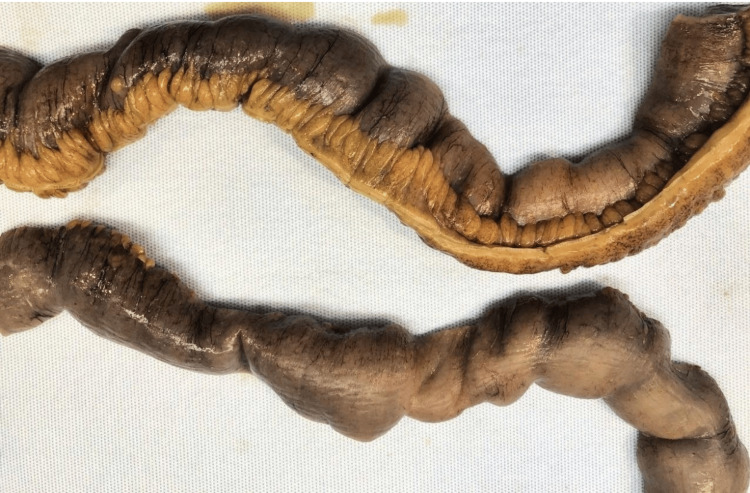
External surface of the intestine and mesentery The serosal surface demonstrates a smooth appearance with characteristic circular fibrotic bands that correspond to the underlying luminal diaphragmatic strictures. The adjacent mesentery appears unremarkable without evidence of inflammation or fibrosis.

**Figure 4:**
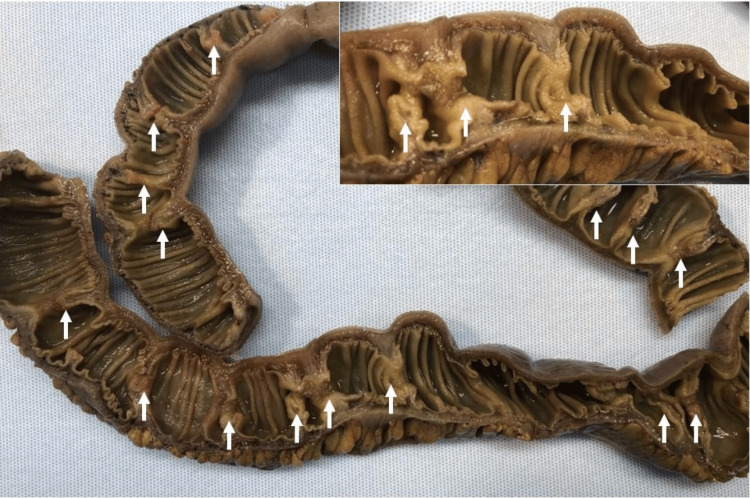
Gross examination of the intestinal lumen The intestinal lumen demonstrates multiple circumferential web-like strictures (diaphragms) resulting in varying degrees of luminal narrowing, ranging from partial to near-complete obstruction. The overlying mucosa at the sites of narrowing exhibits focal erythema and superficial erosions, while the remaining mucosal surfaces appear unremarkable.

**Figure 5:**
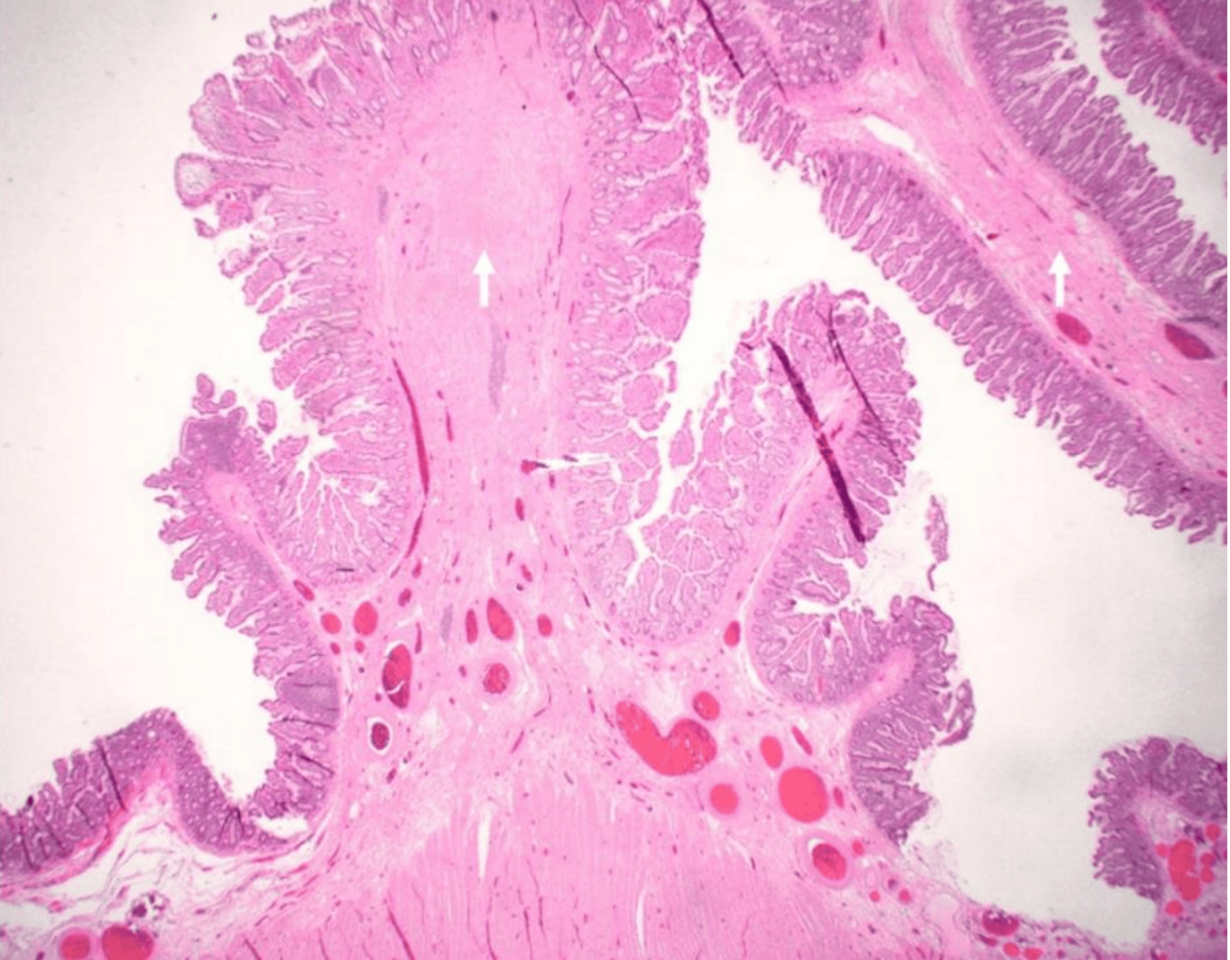
Microscopic examination of the intestine with low magnification (12x) Recurrent mucosal injury results in characteristic extensive submucosal fibrosis (arrow), causing the formation of thin, circumferential diaphragm-like strictures that significantly narrow the intestinal lumen. The muscularis propria is uninvolved.

**Figure 6:**
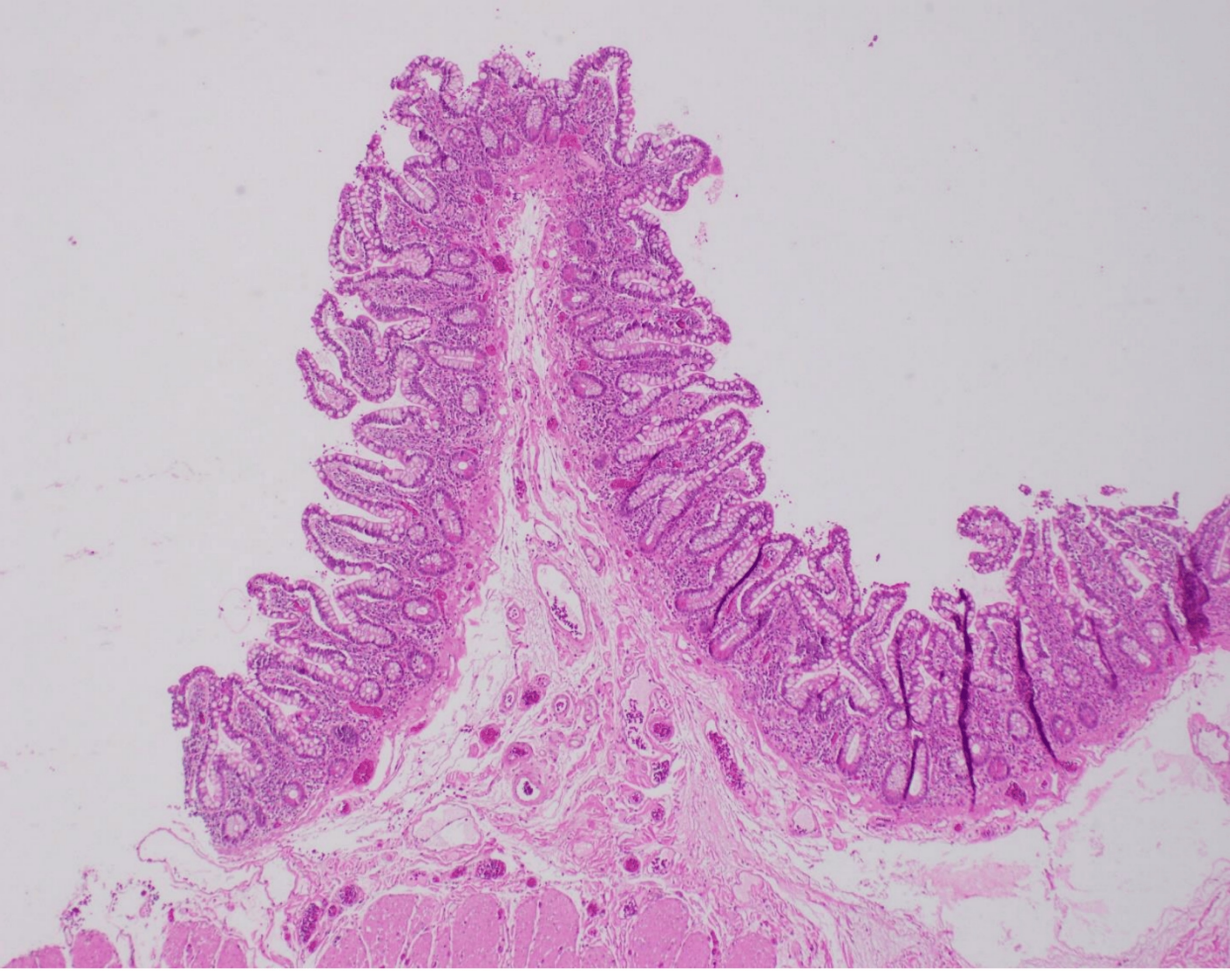
Normal unaffected bowel histology A normal, unaffected wall shows a delicate, thin layer of muscularis mucosa and submucosal adipose tissue. Recurrent injuries and repairs over time result in the replacement of normal architecture with fibrotic scar tissue in the mucosa, extending to the submucosa over time.

**Figure 7:**
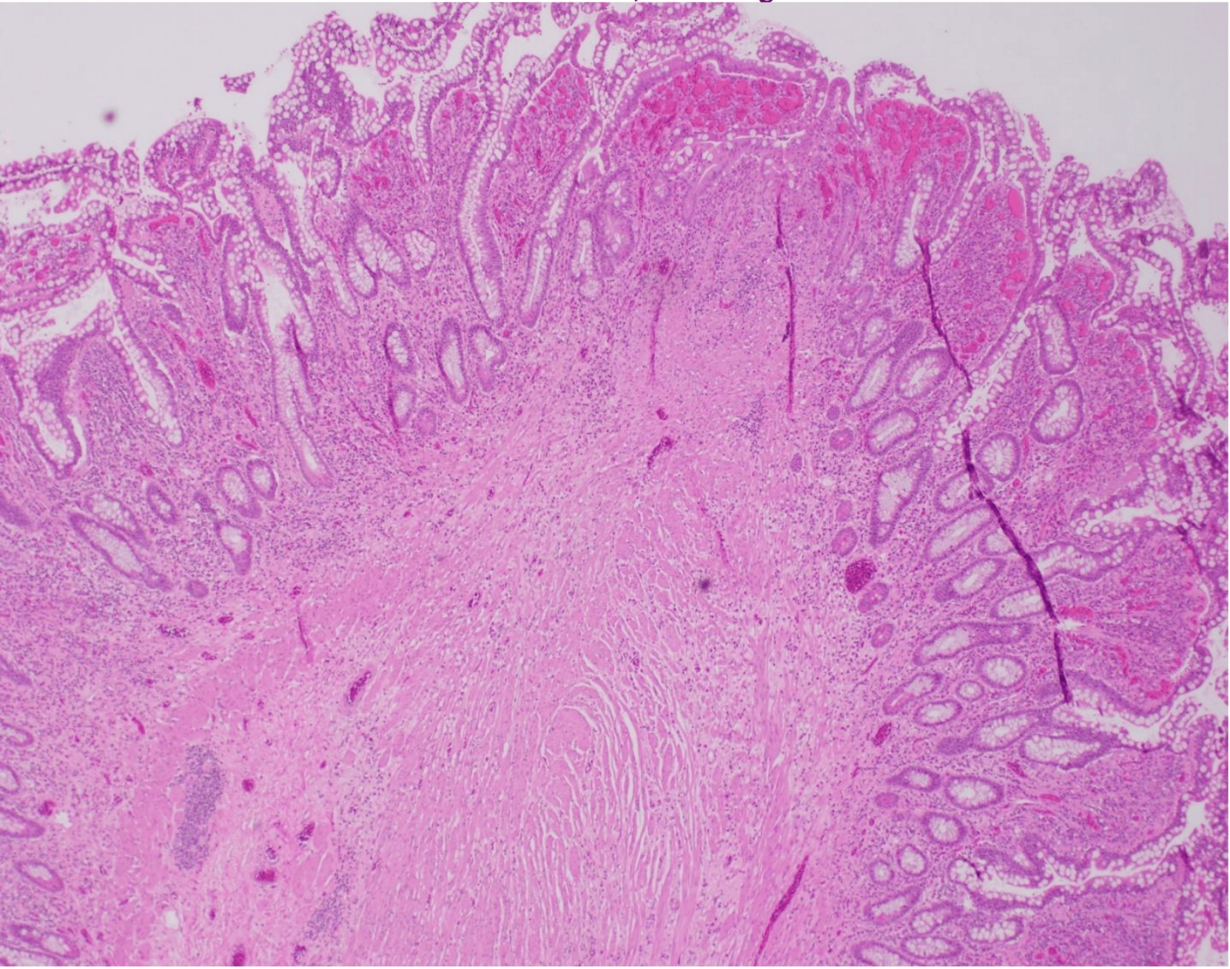
Microscopic examination of the intestine with high magnification (40x) Focal areas of mucosal injury, some with accompanying granulation tissue, are present overlying the narrowed lumen, indicating ongoing or previous mucosal damage.

Gross examination of the resected bowel specimen demonstrated a smooth serosal surface with subserosal intermittent circular fibrotic bands. The mesenteric fat appeared unremarkable (Figure 3). Upon opening the specimen, multiple annular diaphragm-like webs were identified, each exhibiting variable degrees of central luminal narrowing (Figure 4). Microscopic examination revealed characteristic thin, annular fibrotic membranes confined to the submucosa and mucosa (Figure 5). The muscularis propria and the remaining submucosal architecture appeared intact and unremarkable. The mucosa overlying the areas of luminal narrowing demonstrated focal fibrosis, acute inflammation, and reactive epithelial changes (Figure 5). The background mucosa throughout the remainder of the intestinal specimen appeared normal (Figure 6). The inflammatory population and the overall mucosal architecture were otherwise unremarkable (Figure 7). No dysplasia and no microorganisms were identified.

Postoperative course

The nasogastric tube was removed on postoperative day (POD) 2. Flatus was recorded on POD 3, and the first bowel movement on POD 4. Diet was advanced as tolerated; pain was controlled with acetaminophen and low-dose oxycodone. Plain radiography on POD 6 showed resolution of small bowel dilation. The patient was discharged on POD 7 with instructions for lifelong NSAID avoidance. At the two-month surgery clinic follow-up, he was asymptomatic with normal bowel habits.

## Discussion

Diaphragm disease occurs as a consequence of chronic NSAID enteropathy leading to ulceration and reduction of blood flow to the intestinal mucosa, along with systemic prostaglandin depletion, which impairs mucosal defense. Secondary healing through collagen deposition and subsequent mucosal and submucosal fibrosis results in the formation of concentric webs [6,7]. While cyclooxygenase-2 (COX-2) selective inhibitors reduce the risk of enteric injury relative to nonselective NSAIDs via preservation of de novo prostaglandin synthesis, they do not abolish enteric injury as topical mechanisms persist. In one study screening 1,262 symptomatic patients, 156 regular NSAID users were identified, of whom 31 (20%) had NSAID-induced small intestinal injury, of which diaphragm disease represented the severe end of this spectrum [8]. The study also found greater injury with combined aspirin plus non-aspirin NSAID use compared with aspirin alone and other factors that interfere with NSAID metabolism, such as CYP2C9 variants, as potential contributors, with certain polymorphisms linked to diaphragm disease risk.

The ileum is the most frequent site for small bowel diaphragm disease. In up to 92% of patients, standard CT scans typically reveal some abnormalities, with the most prevalent findings being small bowel strictures and thickening of the small bowel wall as well as transition points; however, these findings are largely non-specific and do not aid the diagnostic process [4,9]. CT scans are also largely unable to visualize strictures due to their thickness rarely exceeding 2 mm; in contrast, CT and MR enterography improve luminal distension and spatial resolution that can better visualize the small bowel and lesions such as luminal strictures [5]. A study involving 11 patients with small bowel damage caused by COX inhibitors revealed that CT enterography was useful in identifying small bowel abnormalities in 8 of 11 patients (73%); among these, 5 of 8 patients (63%) had multiple lesions [5]. Despite this, CT imaging may occasionally fail to fully capture the multiplicity of lesions, as demonstrated in this case where more than 20 circumferential fibrous septa were identified over a 36-cm length of the resected intestinal segment, highlighting the added diagnostic value of visualization through endoscopy or surgical exploration.

Capsule endoscopy can be a sensitive alternative in detecting diaphragm disease, provided luminal patency is ensured [10,11]. Using a patency capsule can confirm patency and reduce the risk of capsule retention. Once patency is established, capsule endoscopy characterizes lesion distribution and can guide the route for double-balloon enteroscopy (DBE) that assists with visualization and therapy through dilation of strictures. However, in current practice, DBE is only used judiciously in carefully selected patients. In a study, diagnosis was established after laparotomy in 52.1% while only 41.5% of cases were diagnosed by capsule endoscopy and/or DBE preoperatively, indicating a disconnect between available diagnostics and knowledge surrounding clinical practice [10].

Due to the current diagnostic methodology and practices surrounding diaphragm disease, surgical intervention remains the most employed treatment method, as diagnosis generally occurs during laparotomy for the management of presumed SBO. While cases of high-grade obstruction without patency favor surgical exploration, there remains a large host of cases where less aggressive methods of diagnosis and treatment of diaphragm disease are appropriate. Some cases have documented that isolated discontinuation of NSAIDs has led to disease reversal without endoscopic intervention or resection [9]. In cases where NSAIDs are difficult to discontinue, alternative strategies, including prostaglandin analogues, have been employed to reduce recurrence, yet the only confirmed preventive measure remains NSAID withdrawal [10,12]. Although proton pump inhibitors (PPIs) have traditionally been employed in treating upper gastrointestinal issues associated with NSAID use, a recent meta-analysis indicates that PPIs might elevate the risk of NSAID-induced small bowel enteropathy [13].

Clinicians have a range of diagnostic options at their disposal, including standard CT, CT enterography/MR enterography, capsule endoscopy, and therapeutic DBE as an alternative to laparotomy, each offering distinct risk-benefit profiles. Early diagnostic tools may identify a larger group of patients who may be able to forego more invasive testing and management; however, when obstruction is complete, retention risk is high, or medical therapy has failed, early surgery, as undertaken here, remains appropriate.

## Conclusions

Diaphragm disease should be contemplated in elderly patients taking NSAIDs who present with abdominal discomfort, iron deficiency anemia, and concerns of SBO, particularly when lacking prior surgical adhesions or risk factors. Small bowel-specific imaging paired with endoscopic modalities or timely surgical exploration can make the diagnosis and allow for definitive therapy. Endoscopic balloon dilation, where feasible, and permanent NSAID cessation can potentially prevent bowel resection and morbidity in selected patients. Heightened awareness among clinicians can reduce diagnostic delay and improve outcomes for this under-recognized consequence of chronic NSAID therapy.
